# MicroRNA profile in very young women with breast cancer

**DOI:** 10.1186/1471-2407-14-529

**Published:** 2014-07-21

**Authors:** Maria Peña-Chilet, Maria T Martínez, Jose A Pérez-Fidalgo, Lorena Peiró-Chova, Sara S Oltra, Eduardo Tormo, Elisa Alonso-Yuste, Beatriz Martinez-Delgado, Pilar Eroles, Joan Climent, Octavio Burgués, Jaime Ferrer-Lozano, Ana Bosch, Ana Lluch, Gloria Ribas

**Affiliations:** 1 Medical Oncology and Hematology Unit, INCLIVA Biomedical Research Institute, Av. Blasco Ibañez, 17, Valencia 46010, Spain; 2 Biobank, INCLIVA Biomedical Research Institute, Av. Blasco Ibañez, 17, Valencia, Spain; 3 Pathology Unit, INCLIVA Biomedical Research Institute, Av. Blasco Ibañez 17, Valencia, Spain; 4 Laboratory rare diseases, ISCIII, Sinesio Delgado, 4, Madrid, Spain; 5 Memorial Sloan-Kettering Cancer Center, 417 E. 68th St, New York, NY 10065, USA

**Keywords:** Breast cancer, Young women, miRNA, Molecular profile

## Abstract

**Background:**

Breast cancer is rarely diagnosed in very young women (35years old or younger), and it often presents with distinct clinical-pathological features related to a more aggressive phenotype and worse prognosis when diagnosed at this early age. A pending question is whether breast cancer in very young women arises from the deregulation of different underlying mechanisms, something that will make this disease an entity differentiated from breast cancer diagnosed in older patients.

**Methods:**

We performed a comprehensive study of miRNA expression using miRNA Affymetrix2.0 array on paraffin-embedded tumour tissue of 42 breast cancer patients 35 years old or younger, 17 patients between 45 and 65 years old and 29 older than 65 years. Data were statistically analyzed by t-test and a hierarchical clustering via average linkage method was conducted. Results were validated by qRT-PCR. Putative targeted pathways were obtained using DIANA miRPath online software.

**Results:**

The results show a differential and unique miRNA expression profile of 121 miRNAs (p-value <0.05), 96 of those with a FDR-value <0.05. Hierarchical clustering grouped the samples according to their age, but not by subtype nor by tumour characteristics. We were able to validate by qRT-PCR differences in the expression of 6 miRNAs: miR-1228*, miR-3196, miR-1275, miR-92b, miR-139 and miR-1207. Moreover, all of the miRNAs maintained the expression trend. The validated miRNAs pointed out pathways related to cell motility, invasion and proliferation.

**Conclusions:**

The study suggests that breast cancer in very young women appears as a distinct molecular signature. To our knowledge, this is the first time that a validated microRNA profile, distinctive to breast cancer in very young women, has been presented. The miRNA signature may be relevant to open an important field of research in order to elucidate the underlying mechanism in this particular disease, which in a more clinical setting, could potentially help to identify therapeutic targets in this particular set of patients.

## Background

Breast cancer (BC) is the most common cancer in women worldwide. It is estimated that breast cancer will account for up to 29% of all new cases of cancer in women in the USA in 2013 [1]. Although the median age at onset is 61 years, approximately one in forty women diagnosed with early breast cancer is very young, constituting 5 to 7% of all cancer deaths in these women [2,3].

Breast cancer in very young women is typically more aggressive than in their older counterparts, in part owing to the over-representation of high-grade, triple-negative tumours in the former patients. Indeed, very young patients diagnosed with breast cancer usually present larger and poorly differentiated tumours, lymph node invasion, HER2 overexpression and an absence of hormone receptor (HR) expression [4,5]. It is still a matter of controversy, however, as to whether breast cancer in very young patients (BCVY) is a biologically unique entity that should be considered separately or if its behaviour and outcome is solely due to the higher frequency of more aggressive subtypes. There are contradictory studies on the identification of young age as an independent prognostic factor [5-7].

MicroRNAs (miRNAs) are post-transcriptional regulators that bind to complementary sequences on target messenger RNA transcripts (mRNAs), usually resulting in translational repression or target degradation and gene silencing [8-10]. Deregulation of many of the miRNA’s expression has been linked to various types of disease [11-13]; (http://www.mir2disease.org/). The expression of several miRNAs has been found to be deregulated in some types of cancer [14,15]. High levels of some miRNA have been linked to stem cell promotion [16-18], while others exhibit a reduced expression of many, promoting loss of differentiation [19,20]. Both are common traits in tumour development, but many other unknown underlying mechanisms could be affected [21]. Moreover, miRNA expression profiling has been previously shown to be a useful tool for classifying different cancer risk stratification, outcome prediction and classification of histological subtypes [22,23]. Regarding breast cancer, several studies have identified molecular markers, such as miR-21, miR-9, let-7, miR-205, miR-200 family, miR-126 and miR-335, which correlate to tumours of high metastatic and proliferative capacity, larger size and thus poorer prognosis [18,24-29].

As previously stated, very little is known about the biology of BCVY, and published studies have focused mainly on hereditary tumours and genomic traits [30,31]. Nevertheless, the data from two previous published studies point to the possibility that BCVY could have a distinct molecular identity [32,33].

We performed a study with the aim to identify whether BCVY had a different miRNA profile compared with older age. A secondary objective was to identify those miRNA that are typically up- or downregulated in the tumours from very young patients, which could reveal the ongoing mechanisms of the development of the tumour and of its aggressiveness at an early age.

## Methods

### Sample selection

**Figure 1:**
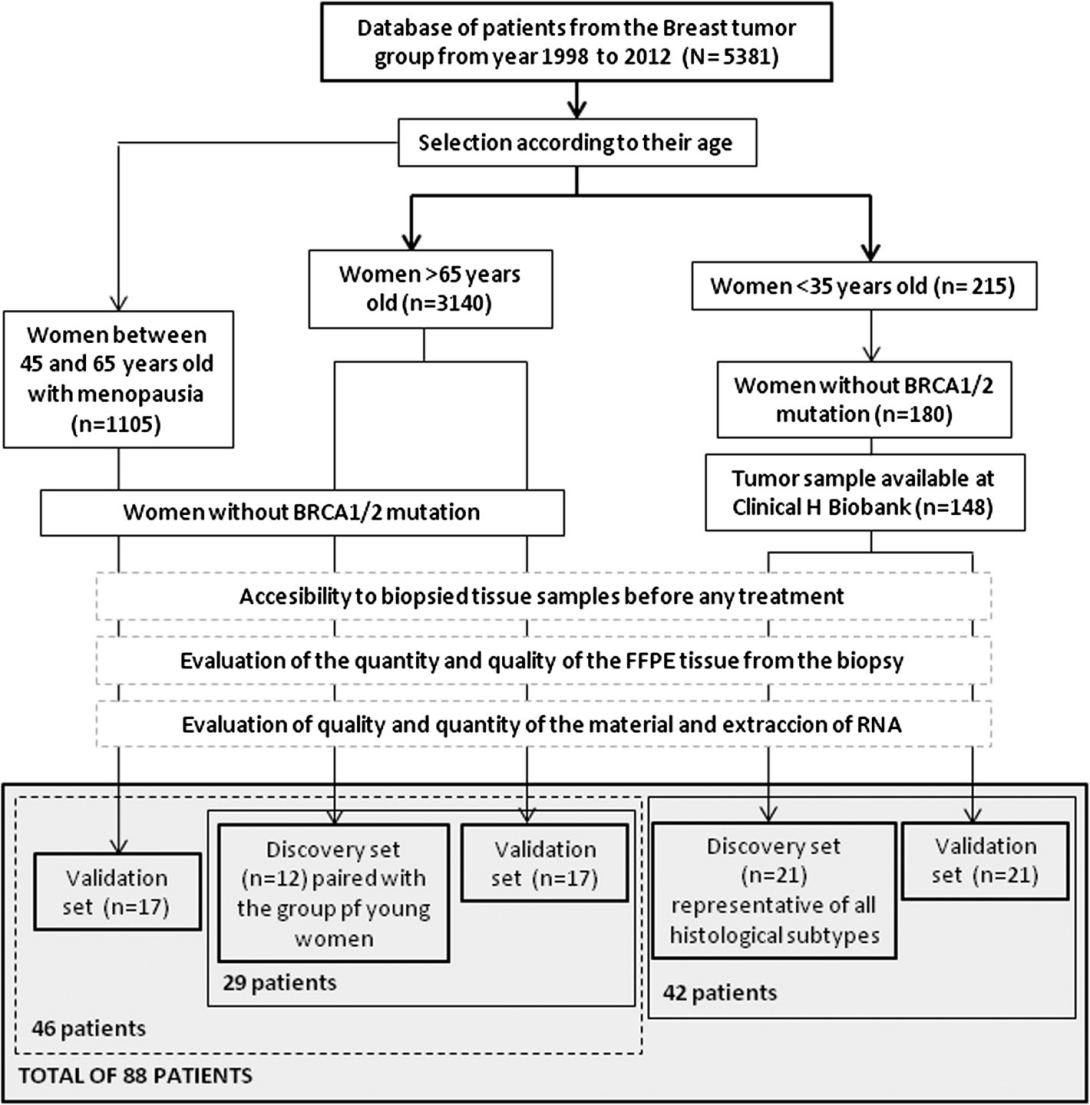
Flow diagram representing the guidelines followed in the selection of the patients suitable for the present study.

Tissue samples were obtained from patients undergoing surgery for breast cancer at the Hospital Clinico Universitario of Valencia from among the Spanish population. As shown in Figure 1, we selected two groups of women according to their age, one group of women 35 years old or younger (BCVY), and the other of those older than 65 years of age. After excluding those samples with known BRCA1/2 mutation, we got a total of 148 women 35 years old or younger and 3140 patients over 65 years of age with an available biopsy sample before any treatment. We screened for BRCA1/2 status according to the ESMO Guidelines for Hereditary Breast Cancer that follow standard criteria for high-risk patients and from those, we excluded the positive cases from our study as well as those with known or suspected family history of breast cancer. All the participants, with one exception, were Caucasian women of European origin and gave written, informed consent by signing an approved Biobank document allowing research use of the remaining diagnostic tumour material. The study was approved by the Institutional Health Incliva-Hospital Clinico Ethics Committee.

After evaluating the quantity and quality of the material, a total of 33 breast cancer patients were included in this study: 21 women diagnosed before the age of 35 categorized as the BCVY group, and 12 breast cancer patients older than 65 years old and matched by tumour subtype (Table 1). We also collected tissue samples from three young cancer-free women undergoing mammary reduction surgery, with ages 28, 34 and 40 years old, and BMI within normal values (between 20 and 24).

**Table 1:** Clinical tumour characteristics of the sample groups used in the present study

	**Discovery set N = 34 (%)**		**Validation set N = 55 (%)**		
**Age group**	<35 (n = 22)*	**>65 (n = 12)**	**<35 (n = 21)**	**45-65 (n = 17)**	**>65 (n = 17)**
Age mean (SD)	31.41 (4.04)	73.3 (10.18)	31.4 (2.87)	56.94 (5.58)	69.4 (4.48)
BMI mean (SD)	21.98 (3.53)	29.44 (6.66)	23.95 (5.50)	30.15 (8.04)	30.23 (6.55)
Histological grade					
I	1 (4.55)	3 (25.00)	3 (14.29)	8 (47.06)	3 (17.65)
II	8 (36.36)	3 (25.00)	7 (33.33)	5 (29.41)	12 (70.59)
III	13 (59.09)	6 (50.00)	11 (52.38)	4 (23.53)	2 (11.76)
Histological Type					
Ductal	19 (86.36)	11 (91.67)	19 (90.48)	13 (76.47)	12 (70.59)
Lobular	0 (0.00)	0 (0.00)	1 (4.76)	0 (0.00)	2 (11.76)
Others	3 (13.64)	1 (8.33)	1 (4.76)	4 (23.53)	3 (17.65)
Tumour size					
< 2 cm	3 (13.64)	9 (75.00)	3 (14.29)	11 (4.71)	14 (82.35)
2-5 cm	15 (68.18)	2 (16.67)	15 (71.43)	6 (35.29)	3 (17.65)
> 5 cm	4 (18.18)	1 (8.33)	3 (14.29)	0 (0.00)	0 (0.00)
Nodal status					
Positive	10 (45.45)	4 (33.33)	7 (33.33)	6 (35.29)	3 (17.65)
Negative	12 (54.55)	8 (66.67)	14 (66.67)	11 (64.71)	14 (82.35)
Receptors					
ER+	17 (77.27)	9 (75.00)	14 (66.67)	14 (82.35)	14 (82.35)
ER -	5 (22.73)	3 (25.00)	7 (33.33)	3 (17.65)	3 (17.65)
PR+	15 (68.18)	7 (58.33)	14 (66.67)	13 (76.47)	13 (76.47)
PR -	7 (31.82)	5 (41.67)	7 (33.33)	4 (23.53)	4 (23.53)
HER2+	10 (45.45)	3 (25.00)	5 (23.81)	2 (11.76)	2 (11.76)
HER2 -	12 (54.55)	9 (75.00)	16 (76.19)	15 (88.24)	15 (88.24)
Ki67					
1-14%	5 (22.73)	3 (25.00)	5 (23.81)	8 (47.06)	5 (29.41)
14-30%	8(36.36)	5 (41.67)	11 (52.38)	7 (41.18)	10 (58.82)
>30%	9 (40.91)	4 (33.33)	5 (23.81)	2 (11.76)	2 (11.76)
Histological subtype					
Luminal A	5 (22.73)	3 (25.00)	3 (14.29)	8 (47.06)	5 (29.41)
Luminal B	6 (27.27)	4 (33.33)	10 (47.62)	4 (23.53)	9 (52.94)
TN	2 (9.09)	2 (16.67)	3 (14.29)	3 (17.65)	2 (11.76)
Luminal/HER2	5 (22.73)	2 (16.67)	2 (9.52)	0 (0.00)	1 (5.88)
HER2	4 (18.18)	1 (8.33)	3 (14.29)	2 (11.76)	0 (0.00)

BMI stands for Body Mass Index and is expressed in terms of kg/m^2^. ER: Estrogen receptor; PR: Progesterone receptor. HER2: ErbB2 receptor; +/−: presence (+) and absence (−) of receptor overexpression. HER2 is considered positive (+) when immunohistochemical analyses show +++/+++ or ++/+++ (and FISH shows HER2 amplification). Subtypes have been categorized according to Hormonal Receptors, HER2 expression and Ki67 value. *One of the initial 22 samples younger than 35 years old, was removed from the study due to methodology QC thresholds.

For the validation set, we included a total of 55 new independent samples from HCUV, selected with the same aforementioned criteria, 21 of which were BCVY patients, 17 were older than 65 years, and 17 individuals were post-menopausal women between 45 and 65 years old. We included this new age group in order to better establish the cut-off age of the miRNA profile. Overall, the present study was performed on a total of 88 breast cancer patients.

All of the tissue samples were biopsied from primary tumour and locked in FFPE (Formalin-fixed paraffin-embedded). Three-μm thick paraffin sections were stained with haematoxylin and eosin (H&E) in order to obtain a histological control of all samples. All tissue samples contained >30% tumour material. Tumour grade was assessed based on Bloom-Richardson scoring system. Images of the sample’s tissues can be seen in Additional file 1.

To obtain the molecular characteristics of the tumour, presence of molecular markers ER, PR, HER2 and Ki67 was evaluated following the ACO/CAP guidelines. ER and PR status were obtained by IHC staining and HER2 Immunohistochemical staining on TMA sections was performed by the EnVision method with a heat-induced antigen retrieval step. Staining results were assessed by a pathologist. ER and PR were scored based on two-stage scoring system: positive (1) for >10% of ER/PR positive cells and negative (0) for less than 10%, as described previously. Proliferation was assessed measuring percentage of Ki-67 expression. HER2 was called positive either by detection of ERBB2 gene amplification by FISH analysis and/or 3+ staining by DAKO system on HercepTestTM. Where duplicate cores gave discordant results, the higher score was used. Breast cancer tumours were classified into four subtypes based on IHC-model (Tang P. et al., 2009) as: luminal A (ER + and/or PR+, HER2−, Ki67 < 14%); luminal B (ER + and/or PR+, HER2-, Ki67 > 14%);, Triple Negative (ER−, PR−, HER2−) and HER2 overexpressed/amplified (ER−, PR−, HER2+), plus an additional group Luminal/HER2 (ER + and/or PR+, HER2+).

### RNA isolation

Total RNA was isolated using RecoverAll Total Nucleic Acid Isolation Kit (Applied Biosystems by Life Technologies, Carlsbad, California, USA) following the manufacturer’s protocol. RNA concentration was measured using a NanoDrop ND 2000 UV–vis Spectrophotometer (Thermo Fisher Scientific Inc., Wilmington DE, USA).

RNA integrity determined by RIN (RNA integrity number) value was assessed with the Agilent 2100 Bioanalyzer using the RNA 6000 Nano Assay (Agilent Technologies Inc., Santa Clara, CA, USA).

### miRNA microarray

Microarray expression profiling was performed using GeneChip® miRNA 2.0 Array (Affymetrix, Santa Clara, CA, USA), containing a total of 15,644 probes, in 11 replicates, including 1100 human mature miRNA, their precursors, 2334 human snoRNA (small nucleolar RNA) and scaRNA (small Cajal body-specific RNA) annotated in the miRBase v.15 database. Hybridisation and scanning were performed according to the Affymetrix standard protocol, using Affymetrix Expression Console software. The microarray dataset is publicly available at GEO database (GSE48088) http://www.ncbi.nlm.nih.gov/geo/info/linking.html.

### Array data processing and analysis

Data passed quality controls implemented in Expression Console software, build 1.2.0.20, and QC Tool (Affymetrix, Santa Clara, CA, USA). All data were normalised by the DABG-RMA, detected above background (DABG) Robust Multichip Average (RMA) method. We selected 1100 human miRNAs, and set as the threshold for low expression the highest common intensity value of miRNA from vegetable organisms (not supposed to be expressed in humans) included in the microarray. After filtering the data, in order to determine the differences in expression pattern between tumours from BCVY patients and from older ones, we carried out differential expression analyses with the POMELO II tool (http://asterias.bioinfo.cnio.es). We performed a *t*-test by permutation testing, and p-values were adjusted for multiple comparisons by Benjamini & Hochberg False Discovery Rate (FDR). Those miRNAs with a FDR p-value <0.05 were considered statistically significant. Average linkage hierarchical clustering was performed to obtain clusters of data sets, using Gene Cluster and Treeview software (http://www.eisenlab.org/eisen/).

In order to evaluate whether the miRNA profile detected was a result of age differences or if it was influenced by confounding factors such as tumour size, histological grade and other features characteristic of more aggressive tumours, we performed a *t*-test analysis on the filtered miRNA expression data after correcting by the influence of nodal status, histological grade, percentage of ki67 expression and tumour size. Data were adjusted obtaining the correlation between miRNAs’ expression and tumour characteristics via linear regression with R Studio (http://www.rstudio.com/), using the remaining residuals for the correction.

### Validation by qRT-PCR

Quantitative real time-PCR (qRT-PCR) of selected miRNAs was performed on breast tumour tissue samples in independent series of women younger than 35 years old, women between 45 and 65 years old, and women older than 65 years using TaqMan microRNA Assays (Applied Biosystems by Life Technologies, Carlsbad, California, USA). Normalisation was done with RNU43 snoRNA. The data were managed using the Applied Biosystems software RQ Manager v1.2.1. Relative expression was calculated by using the comparative Ct method and obtaining the fold-change value (ΔΔCt). Data analyses were performed via GraphPad Prism v6.00. The ANOVA test was performed, and Tukey’s test was used to correct for multiple comparisons (p-value threshold of 0.05). Brown-Forsythe test was used to assess the homogeneity of the variances in the different samples groups. Finally, the association of the validated miRNAs was adjusted by breast cancer subtypes using RStudio (http://www.rstudio.com/).

### Pathway enrichment analysis and candidate gene searching

DIANA miRPath pathway enrichment analysis was used to gain insight into global molecular networks and canonical pathways related to differentially expressed miRNAs (http://diana.imis.athena-innovation.gr/DianaTools/index.php?r=mirpath/index). The software performs an enrichment analysis of multiple miRNA target genes comparing each set of miRNA targets to all known KEGG pathways. Those pathways showing a FDR p-value <0.05 were considered significantly enriched between classes under comparison. We also searched for candidate genes using Target-scan online software (http://www.targetscan.org/) and previously published data.

## Results

### miRNA expression profiling in primary breast tumours from discovery set

**Figure 2:**
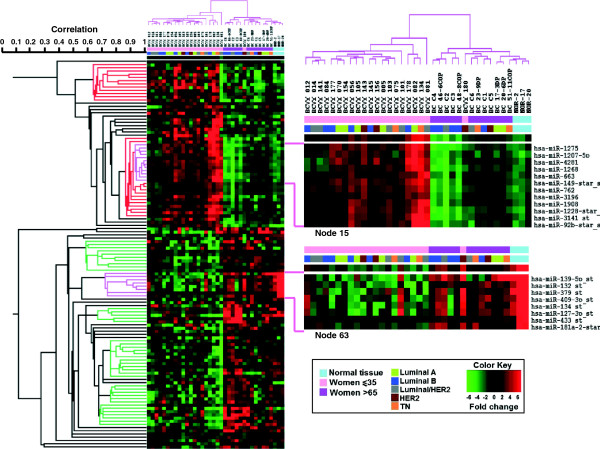
**Heatmap representing an unsupervised cluster performed according to a hierarchical clustering method centered on the median, using differently expressed human miRNA expression values from an Affymetrix array assay.** The miRNAs plotted are those which gave significant FDR p-values after performing a *t*-test according to the age group of the samples. Correlation values (0–1) of different sub-nodes are shown in the figure according to data obtained from Cluster and Treeview software. The two most correlated, overexpressed-sub-nodes are highlighted in red (from top to bottom, sub-node 92 and sub-node 29); highlighted in green are the most correlated repressed sub-nodes (from top to bottom 102, 79, 52, 54 and 73). The selected nodes for validation are shown in pink (node 15 and node 63).

After initial pre-processing of 1100 miRNA expression data, we discarded miRNAs with uniformly low expression, reducing the number of miRNA to a total of 251 hsa-miRNAs. Finally we detected 119 miRNAs differentially expressed between the two groups (p-value < 0.05) and after adjusting for FDR, 96 miRNAs remained significant (Additional file 2). In addition, all of these miRNA’s expression remained significantly different even after correcting by possible confounding factors (tumour size, nodal status, ki67% and histological grade), suggesting that young age is an independent factor (Additional file 3).Mammary tissue from three cancer-free women was included as healthy control group. When unsupervised hierarchical clustering was performed two clearly different two clearly different groups of samples (Figure 2). One group included all except one of the BC tissue samples from very young women. The other group contained all the samples from both older women and normal tissue. We were able to identify two major nodes of differentially regulated miRNA in the BCVY group. As shown in Figure 2, the 52 miRNAs overexpressed in the BCVY group can be seen clustered in the top node, 115, (correlation 0.383) while the lower node, 119, shows the 67 miRNAs found to be repressed in this BCVY set of tumours (correlation 0.102).

From the overexpressed node (node 115) we were able to identify two differentiated sub-nodes: node 92 (correlation value 0.63) and node 84 (correlation value 0.66), that appears to be more homogenous inside each group of patients. Within node 84 we selected sub-node 15 (correlation 0.923) including miRNAs with the highest significant expression difference between Young *vs* Old women-Normal. When scanning the repressed node 119, we identified six interesting sub-nodes, covering most of the node: sub-node 102 (correlation 0.582), sub-node 79 (correlation 0.667), sub-node 76 (correlation 0.678), sub-node 63 (correlation 0.72), sub-node 54 (correlation 0.75) and sub-node 52 (correlation 0.75). While sub-nodes 54 and 52 reached the highest correlation value, we selected sub-node 63 for the further study, since the expression of the whole cluster in cancer-free tissue samples was remarkably high, and also because the BC patients showed levels uniformly higher than the BCVY ones. MicroRNAs from the selected sub-nodes are listed in Figure 2, and their expression fold change and p-values are shown in Additional file 4.

Regarding the hsa-miRNA-star (hsa-miR-92*, hsa-miR-149* and hsa-miR-1228*), when the main form of both miRNA species was also differentially expressed (hsa-miR-92* and hsa-miR-149*), we selected the main form of each miRNA (hsa-miR-92-3p and hsa-miR-149-5p) in order to get more reliable results.

### Pathway enrichment and selection of candidate miRNAs for validation step

With the 20 selected miRNAs, composing the two most striking sub-nodes, we performed in silico analyses and database searches to determine which ones might be more biologically relevant in the breast cancer context, and eligible for validation. Considering that the miRNAs target from small to a very large number of mRNA transcripts, common deregulation of a set of miRNA could have a potential impact on various biological pathways. To asses which pathways could be affected by the deregulation, we used DIANA miRPath v2.0. When studying miRNAs from the selected sub-nodes, KEGG pathway enrichment analysis revealed several pathways overrepresented with a FDR p-value <0.05, including adhesion and mobility related pathways (extracellular matrix-receptor interactions, glycosaminoglycan biosynthesis, adherens junction, focal adhesion, cell adhesion molecules, regulation of actin cytoskeleton), biological proliferation-or differentiation-related pathways relevant in cancer development or progression (MAPK signalling, endocytosis, axon guidance), circadian rhythm and several cardiomyopathies . Within the genes involved in the pathways related with the selected miRNAs, we found many integrins, laminins, collagen, MAPkinases, semaforins, interleukins, claudins and calmodulins, among others. More information and FDR p-values associated with each pathway are shown in Additional file 5.

We have finally selected 12 miRNAs (7 from the sub-node 15 and 5 from sub-node 63) as putative candidate targets to perform further studies according to their significance, previous published studies, and implications in biological pathways: hsa-miR-1275, hsa-miR-1207-5p, hsa-miR-149*, hsa-miR-762, hsa-miR-3196, hsa-miR-1228*, hsa-miR-92*, hsa-miR-139-5p, hsa-miR-132, hsa-miR-379, hsa-miR-409-3p, and hsa-miR-433. Supplementary information is available in Additional file 6.

### qRT-PCR validation in an independent set of samples

We performed a validation of the 12 selected miRNAs using qRT-PCR, on an independent set of samples with similar characteristics as those used in the Discovery phase. However, in the validation set, we classified the 55 samples into three groups (21 tumours from women younger than 35 years, 17 tumours from women aged between 45 and 65 and finally 17 tumours from women older than 65) (see the clinical characteristics for the entire sample set in Table 1).

**Figure 3:**
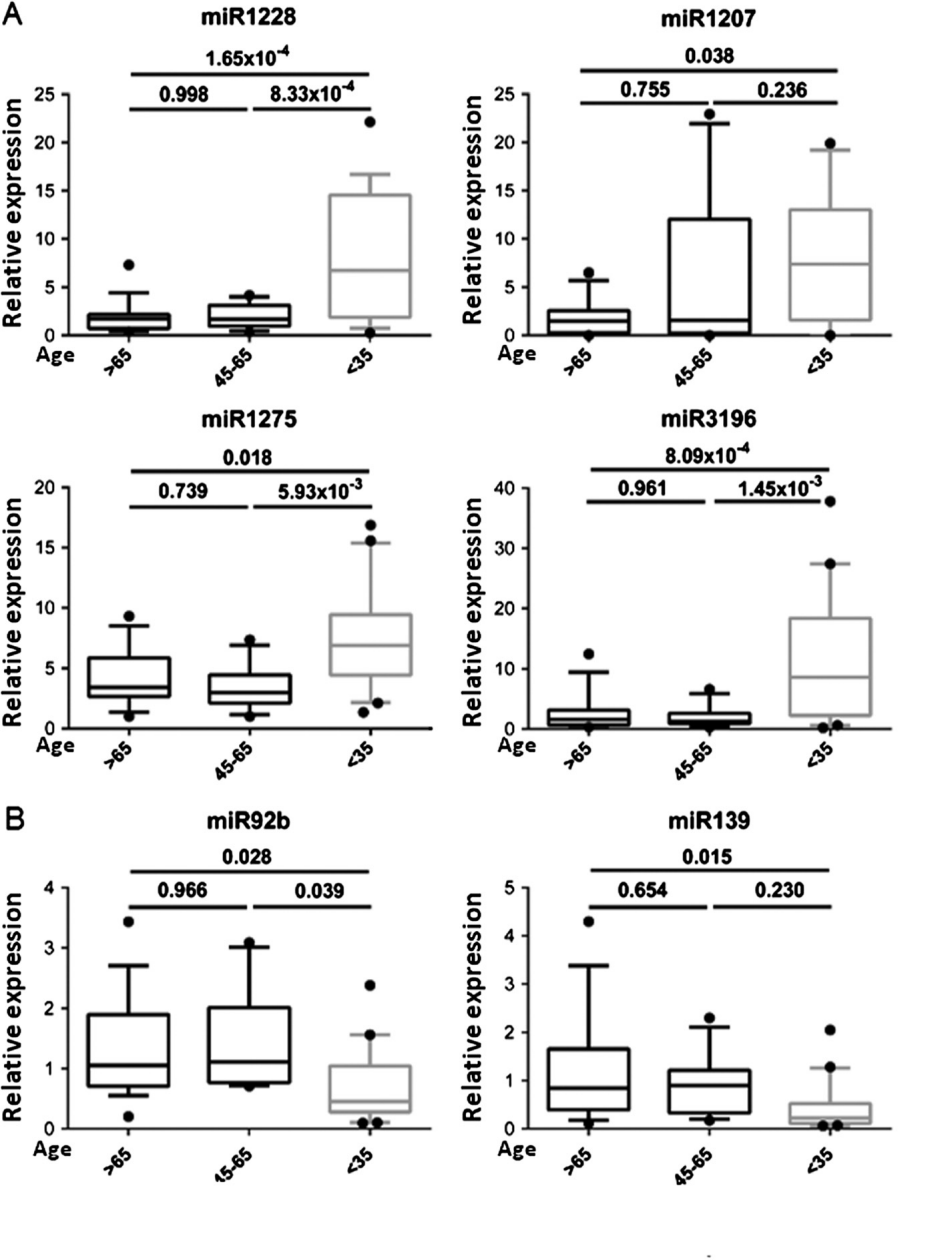
**Relative expression of the validated miRNAs which have significantly different expression in BCVY tumour samples. A**: miRNAs with increased expression in women ≤35 years old (BCVY). **B**: miRNAs with decreased expression in BCVY. Measures of the expression were quantified using the qRT-PCR technique and calculated using the ΔΔCt method. Boxes represent the sample distribution with the mean, vertical lines mark the 10^th^ percentile, and outliers are represented as dots. P-values are calculated via One-Way ANOVA, and Tukey’s method was implemented as a correction for multiple comparisons. The top p-value represents differences between those ≤35 (BCVY) and >65; the bottom-left represents differences between those >65 and those 45–65 (middle group, between 45 and 65 years old); and bottom-right, the differences between those 45–65 and those ≤35 (BCVY).

We did not find evidence suggesting than women older than 65 had any differential characteristics with the 45 to 65 year age group in terms of miRNA expression (Figure 3). Six of the twelve selected miRNAs were confirmed to have a differential expression across groups: miR-1228* (p-value 4.73×10^−5^), miR-3196 (p-value 1.98×10^−4^), miR-1275 (p-value 2.86×10^−3^), miR-92b (p-value 0.012), miR-139-5p (p-value 0.018), and miR-1207 (p-value 0.047). After Tukey’s correction for multiple comparisons, we obtained p-values between each group, confirming that the differences were higher between BCVY samples and the other two older groups (Figure 3). The first four values agree with an upregulation in BCVY, while the last two, miR-92b and miR-139-5p, were indeed downregulated in young women group (see Figure 3 and Additional file 2). Afterward, a *t*-test analysis was used to compare the BCVY group with the two older patient groups, treating the last two as only one group. The results obtained were the following: miR-1228 (p-value 7.13×10^−6^), miR-3196 (p-value 3.23×10^−5^), miR-1275 (p-value 7.77×10^−4^), miR-92b (p-value 2.94×10^−3^), miR-139-5p (p-value 6.57×10^−3^), and miR-1207 (p-value 0.523). After adjusting for tumour subtype, four of them remained statistically significant: miR-1228 (p-value 0.0032), miR-1275 (p-value 0.0010), miR-3196 (p-value 0.0095), miR-139 (p-value 0.0172). It is worth mentioning that all of the other miRNAs tested did indeed lose expression, as was expected; however, no significant value could be obtained due to a lack of detection sensitivity.

### Putative functional implication of the validated miRNAs

In order to establish a possible underlying biological implication with the validated deregulated miRNAs (hsa-miR-1228*, hsa-miR-3196, hsa-miR-1275, hsa-miR-1207-5p, hsa-miR-92b, hsa-miR-139-5p), we used online software and miRNA databases to perform an exhaustive search for enrichment of candidate pathways and genes. Moreover, we text-mined all published studies related to the six validated miRNAs with the terms cancer and/or putatively target genes. We found several enriched pathways related to cell motility and invasion: adherens junction (FDR p-value 1.03×10^−4^), glycosaminoglycan biosynthesis (FDR p-value 2.23×10^−3^), cell adhesion molecules (CAMs) (FDR p-value 0.035) and several related genes such as integrins, claudins and cadherins. We also found enriched pathways involved in proliferation and apoptosis: calcium signalling (FDR p-value 0.035) and Wnt signalling (FDR p-value 0.041) (Table 2).

**Table 2:** Significantly enriched signalling pathways associated to the validated differentially expressed miRNAs

**KEGG Pathway**	**FDR**	**Number of miRNAs**	**Putative target genes**
Adherens junction	1.03×10^−4^	4	*ACTB, WASL, RAC2, IGF1R, PTPRF, TJP1, CDH1, CSNK2B, FARP2, PTPRJ, SSX2IP, PTPRB, PVRL1*
Steroid biosynthesis	1.49×10^−4^	2	*SC5DL, DHCR24, CYP27B1*
Glycosaminoglycan biosynthesis - chondroitin sulfate	2.23×10^−3^	2	*CHPF*
Dilated cardiomyopathy	2.23×10^−3^	4	*ACTB, ADCY1, ITGB8, ITGB3, CACNG7, TTN, ADCY3, ITGA5, ITGB5, DMD, PRKACA, CACNA1C, CACNA2D2, IGF1, CACNA2D1, CACNA1D, PRKACB*
Lysine degradation	0.014	3	*PLOD3, ASH1L, SETD8, MLL2, MLL, SETD1A*
Taste transduction	0.014	4	*GNG13, SCNN1B, GNB1, PRKACA, SCNN1A, PRKACB*
Cell adhesion molecules (CAMs)	0.035	3	*NEGR1, ITGB8, MPZ, SDC3, CLDN19, CLDN11, PTPRF, CDH1, CD276, NRXN2, CD86, ICOSLG, NFASC, PVRL1, NLGN3*
Calcium signaling pathway	0.035	4	*PRKCA, ADCY1, ATP2B2, BDKRB2, ADCY3, ITPKB, SLC8A2, PDE1B, CACNA11, CAMK2A, NOS1, PRKACA, NTSR1, CACNA1C, PHKA1, MYLK3, CACNA1D, GRIN2A, PRKACB, CHRM1*
Fc gamma R-mediated phagocytosis	0.036	2	*PRKCA, WASL, RAC2, PLA2G4F, ASAP3, GSN, FCGR1A, MARCKS, PIP5K1A, PLD2, AKT3, VASP, MYO10, ARF6*
Hypertrophic cardiomyopathy (HCM)	0.036	4	*ACTB, ITGB8, ITGB3, CACNG7, TTN, ITGA5, ITGB5, DMD, CACNA1C, CACNA2D2, IGF1, CACNA2D1, CACNA1D*
Arrhytmogenic right ventricular cardiomyopathy (ARVC)	0.039	3	*ACTB, DSC2, ITGB8, ITGB3, CACNG7, ITGA5, ITGB5, DMD, CACNA1C, CACNA2D2, CACNA2D1, CACNA1D*
Wnt signaling pathway	0.041	4	*PRKCA, DVL3, WNT16, ROCK1, RAC2, VANGL1, PPP2CA, PPP2R5C, CAMK2A, WNT6, PRICKLE1, PPP2R5B, JUN, APC2, PPP2R1A, PRKACA, VANGL2, CSNK2B, WNT9A, PRKACB*

FDR refers to p-values adjusted by Benjamini & Hochberg’s False Discovery Rate. Number of miRNAs is the count of miRNAs involved in the pathway and targeting one or more genes of the pathway.

## Discussion

Our data lead us to think of a differentiated miRNA signature in the group of patients diagnosed at ≤35 years. We have detected a wide range of differentially regulated miRNAs in BCVY when compared to tumours diagnosed in older women and healthy breast tissue. The fact that the older women’s miRNA profile was highly similar to the healthy breast tissue profile (obtained from women under 40 years old) highlights the distinctiveness of the miRNA profile being characteristic of BCVY patients. These differences were maintained after validation, in an independent series of samples using a different technique, in which controls between 45 and 65 years were included, suggesting that our findings are consistent and not a result of the false positives characteristic of high throughput technologies. Although from a limited sample size, the in silico searches done, pointed out to pathways closely related to high cell proliferation, motility and invasion, which support the fact that tumours from young woman have an aggressive behaviour and are prone to metastasis. Other studies in breast cancer showing miRNA profiles characterized by bad prognosis, revealed similar signalling deregulated pathways [34].

Recently, miRNA profiling has arisen as a major approach study technique, that aims to gain more insight into tumour biology, and widespread deregulated miRNA has been demonstrated in various tumour types [35]. It has been shown that specific miRNA, such as miR-195 and let-7a, may play a role as a potential diagnostic tool and a new biomarker for both detecting non-invasive, early breast cancer as well as monitoring treatment effectiveness [36,37]. Furthermore, other studies have revealed miRNA expression to be specific for each breast tissue type, but it seems not to exist a specific miRNA profile distinctive of BC subtypes, which may point to both differences in the biology of tumours, as well as to patterns of response to treatment [38-44]. As suggested in previous studies, our results reinforce the applicability of the study of miRNA expression in FFPE samples, since the small size of the miRNA preserves them from degradation [45]. In addition, the use of Taq-Man-assay for microRNA expression profiling and validation in FFPE breast tissue samples is reported to offer excellent inter-experimental reproducibility and biological accuracy [41,46].

Several studies confirm that BC in very young patients is a more aggressive disease given the high frequency of adverse prognosis factors or more aggressive subtypes at this age [4]. In most studies published the cut-off age for defining young women is 45 years. However, results from a large Korean breast cancer study with 9885 pre-menopausal patients detects a sharp increase in risk of death from breast cancer in women younger than 35 years old, establishing this age as the more reasonable cut-off defining young age-onset breast cancer [47]. Following this recommendation, we have determined 35 years old as the threshold for the young group, since we considered that, despite this cut-off makes harder patient’s recruitment within the young age group, it identifies a more homogeneous population.

Whether breast cancer in the young is a distinct biological entity or just a reflection of a higher percentage of cases with aggressive phenotypes (that are otherwise indistinctive from aggressive tumours in older patients) remains a matter of controversy and an important outstanding question with possible therapeutic implications [6].

In order to minimize clinical-pathological putative confounding effects, we have performed several comparisons in the expression of these miRNA in terms of tumour size, axillary node involvement, histological grade and ki67, with the results showing no significant differences. This absence of significant differences, having considered the most relevant adverse prognostic factors, suggests that young age could be indeed a factor independent of subtype, size, nodal involvement, or proliferative characteristics that merits further study.

Moreover, no distinct therapy is considered for young or very young patients, but in many cases these patients are over-treated as a consequence of their young age. Thus identification of new prognostic or diagnostic platforms and identification of specific targets is needed for this subgroup [48].

Data about genomic profile in young breast cancer patients are scarce in the literature; however, Colak and cols. have recently published an age-specific gene expression signature in breast cancer [33]. Although, the study focused on women younger than 45, it also included 6 samples from those younger than 35 (BCVY). The study pointed to genes barely implicated in the pathways found in our study. That notwithstanding, the authors proposed that there is a different molecular profile characterizing breast cancer in very young women.

A recent retrospective study analysed data from 20 data sets and assessed the role of these genetic signatures in predicting the prognosis in breast cancer in young women. This study confirmed that BC patients aged 40 or less were diagnosed more frequently with oestrogen receptors and HER2 negative tumours. Furthermore, although proliferation-related gene signatures were not associated with age in this study, in two independent cohorts BC in younger patients was associated with immature epithelial cell and growth factor signalling pathways, leading to the conclusion that BC in young women seems to be a distinct entity beyond the intrinsic breast cancer subtype classification [32].

The data obtained with our miRNA profiling supports the evidence proposed by Yau and cols that could be detected an age-dependent signature in BC with a number of samples from young women very similar to ours. Although the conclusions are raised on gene expression analyses the signaling pathways detected including cell cycle, mammary gland development and extracellular matrix (ECM) are also highlighted in our study [49].

In our study, the most significantly set of deregulated miRNAs in BCVY pointed out to pathways related to apoptosis, cell motility, proliferation, mitotic regulatory processes and the PI3K and IGFR transduction that confer tumours high metastatic capacity, increasing progression and invasion (Table 3). Furthermore, many of the pathways involved include integrins and laminins as target genes, described as proliferation genes portraits of breast tumours [50].

**Table 3:** Published information about validated microRNA target genes and implication in cancer

**miRNAs**	**Gene**	**Target/Function**	**Published papers**
miR-1228	*MOAP1*	*Bcl* homologous. When repressed, allows cells to escape from apoptosis.	Yan et al. Apoptosis. 2012 [69]
miR-3196	*PAX2, THTPA, PIK3R2, BBC3*	***PAX2*.** Implicated in suppression of translation (through WT1). Associated to low breast cancer risk. Repression of *PAX2* would promote a more aggressive breast cancer. ***THTPA***. Metastasis tumour suppressor. ***PIK3R2*.** Proliferation pathway. Anti-apoptotic. ***BBC3*.** Pro-apoptotic gene and associated to tumour size.	Beauchemin et al. Molecular Cancer. 2011 [53]. Kovacevic et al. Biochem Biophys Acta. 2008 [58]. Tajnik et al. Cancer Biomarkers. 2012 [66]. Wong et al. PlosOne. 2012 [68]
miR-1275	*IGF1, NFIX, Claudin11*	***IGF1*** related to tumour proliferation. ***NFIX*** hypermethylated in breast cancer lines. **Claudin 11**, cellular adhesion molecule, associated to invasion and capacity of metastasis when downregulated.	Castaño et al. Cancer Discovery. 2013 [54]. Awsare,et al. Oncology reports. 2011 [51]. Webb et al. BMC Cell Biol. 2013 [67]. Özata et al. Endocr Relat Cancer .2011 [62]. Lian et al. Int J Oncol. 2012 [60]. Katsushima et al. J Biol. Chem. 2012 [57]
miR-1207	*DHCR24,* Claudins	***DHCR24*** expression decreased in metastatic prostate cancer. **Claudins.** Associated with cell motility and tumour invasion and spread of tumour cells and metastasis.	Romanuik et al. Am J Pathol. 2009 [64]. Webb et al. BMC Cell Biol. 2013 [67]
miR-139	*RAP1B, c-FOS, IGF1R, TOP2A CXCR4*	***RAP1B***, family RAS (oncogene). It has a pseudogene. ***IGF1R*** Low expression of miR in colo-rectal cancer is associated to more advanced tumours and less survival. **FOS** proteins are implicated in cell cycle, differentiation and cellular transformation. Dowregulation induces increase in apoptosis and more cellular differentiation. **TOP2A**, topoisomerase. Upregulation is associated to tumour proliferation. **CXCR4**, associated with progression and metastasis in CRC. Regulated by HER2-CD44 via miR139.	Guo et al. Cell Biology. 2012 [56]. Milde-Langosch et al. Breast Cancer Res Treat. 2013 [61]. Shen et al. Biochemical Pharmacology. 2012 [65]. Fan et al. Cell Biochem Funct. 2012 [55]. Bao et al. Gastroenterology. 2011 [52]
miR-92b	*PSMD10, FOX2*	***PSMD10*.** Inhibition of the protein will slow down tumour progression in hepatocarcinoma. High increased expression related to worse prognostic in glioma. **FOX2.** RNA-binding protein regulates alternative splicing.	Leidner et al. PlosOne. 2013 [59]. He et al. Nature. 2005 [15]. Qian et al. Gastroenterology. 2012 [63]

Genes targeted by miRNAs were obtained neither via in silico predictions with Targetscan or searching for previously experimental published works. Most relevant genes are included in the table and most interesting cancer-related pathways or mechanisms has been added to *function* column.

If we take into account the set of validated miRNAs (upregulated miR-1228*, miR-3196, miR-1275, miR-1207 and downregulated miR-139-5p and miR-92b) we were able to detect in the literature their implications in escaping of apoptosis and therefore, contributing to migration and invasion [15,51-69]. Downregulation of one particular miRNA, miR-139, might promote gastric tumour progression and metastasis via upregulating CXCR4, Bao and cols suggest that miR-139 might be suppressed by upstream HER2 signaling, a strongly deregulated pathway in breast cancer [52].

We cannot fully reject an influence of demographics or epidemiological factors inherent to young women in the tumour profile obtained. Bearing this in mind, we believe that the inclusion of healthy young breast tissue in the analysis might have minimized these putative confounding epidemiologic aspects.

Oestrogen levels are an age-dependent factor increasing the risk of breast cancer, which can themselves modulate the expression of several miRNAs. Moreover, miRNAs can as well regulate oestrogen receptor levels [70,71]. None of the miRNAs described in the literature related to oestrogen appears to be deregulated in the BCVY profile presented. The different profile obtained between BCVY and normal breast samples from young women (with similar oestrogen levels) supports the idea that the intrinsic levels of oestrogen are not responsible of the miRNA deregulation.

Early pregnancies and lactation are considered protector factors in BC and they could bias the comparison between young and older woman. In our series, older women in general have early age at first birth and still have developed breast cancer, while in the young women we have representation of both circumstances and all of them fall in the same miRNA profile, highlighting young age as the main leading factor of the profiling.

Mammary density is the strongest risk factor for non-familial breast cancer among women, apart from older age. Young women present denser breast tissue in mammographies, which makes more difficult the diagnosis. In addition, differences in normal breast microenvironment seem to exert an influence on the behavior of breast cancer cells in premenopausal women [72]. A recent molecular profiling study performed on adjacent tissue of breast tumours, classifies extratumoral stromal microenvironments into two primary gene expression phenotypes (Active and Inactive). The Active subtype has high expression of genes involved in activation of fibrosis, cellular movement, increased TWIST expression, and positive expression of TGF-b signatures. Inactive phenotype is overrepresented by samples from young women and according to Perez and cols, means higher levels of cell adhesion and cell–cell contact genes [73,74]. The miRNA profile presented in our study pointed out to pathways also related with cell motility and adhesion, however the upregulation of implicated miRNAs indicates a downregulation of the targeted genes and therefore, stroma that correlates with young and denser breast tissue is not the driver in our deregulated miRNAs signature.

Finally, BMI (Body Mass Index) has been recently related to BC risk and survival. In older women higher BMI results in elevated risk of developing BC, while in young women is a protector factor [75]. In our study the mean value of BMI in BCVY is within normal ranges, which makes difficult any speculation about its role in BCVY.

In our study all patients but one are confirmed to be of European Caucasian ethnicity. This implies that our miRNA profile might not be representative of other genetic backgrounds such as African descent, where exists a higher proportion of breast cancer in young women. On the other side, the study has been performed in a very homogeneous population which reinforces the conclusions obtained and we encourage the replication of our findings in other ethnicity breast cancer collections.

## Conclusions

Our results suggest that breast cancer in young patients appears to be a different biological entity. Previously published works and in silico enrichment analyses of the miRNAs deregulated in young women suggest that targeted genes involved in proliferation pathways, cell adhesion, apoptosis, extracellular matrix and cell motility, deregulation of these pathways may lead to more aggressive, proliferative and metastatic tumour phenotypes. A more detailed analysis of those miRNA significantly up- or downregulated could guide both to the establishment of a different hypothesis about the potential molecular mechanisms involved in the carcinogenesis and to identify more specific therapeutic targets in this particular set of patients.

## Abbreviations

ANOVA: Analysis of variances; BC: Breast cancer; BCVY: Breast cancer in very young women; BMI: Body mass index; BRCA1/2: Breast cancer gene 1/2; cDNA: Complementary DNA; Ct: Mean cycle above threshold; DABG-RMA: Detected above background-robust multichip average; FDR: False discovery rate; FFPE: Formalin fixed-paraffin embedded; H&E: Hematoxilin and eosin; HCUV: Valencian Clinic-University Hospital; HER2: Human epidermal growth factor receptor 2; HR: Hormone receptor; Hsa: Homo sapiens; IGFR: Insulin-like growth factor receptor; MAPK: Mitogen-activated protein kinase; miR: microRNA; miRNA: microRNA; mRNA: messenger RNA; PCR: Polymerase chain reaction; PI3K: Phosphoinositol-3-kinase; qRT-PCR: Quantitative real time-PCR; RIN: RNA integrity number; RNA: Ribonucleic acid; RNU43: Ribonucleoprotein; scaRNA: Small cajal-body RNA; snoRNA: Small nucleolar RNA; Wnt: Wingless-type MMTV integration site family.

## Competing interests

The authors declare that they have no competing interests.

## Authors’ contributions

MPC processed the material and extracted RNA, prepare samples for Affymetrix array, performed qRT-PCR, acquired and analyzed all the data and performed statistical analyses, carried out databases and literature searching and contributed in the drafting of the manuscript; MTM performed the selection of patients suitable for the study, helped processing the samples, gave intellectual support, performed literature searching and contributed writing the manuscript; JAPF conceived the study, participated in the patients selection, contributed in the interpretation of the results and in the manuscript writing; LPC processed samples, optimized the used technique and acquired data, also provide useful information and intellectual content to the manuscript drafting; ET, PE and JC helped in the sample processing, interpretation of the results and revised final manuscript; BMD helped in the data analyses, interpretation of the results and in drafting the manuscript; EA, OB and JFL performed the pathological analyses, characterized the tumour tissue samples and performed immunohistochemical techniques, also provide the FFPE material and provide intellectual content and information for the manuscript drafting; AB has been involved in revising the manuscript critically for important intellectual content; AL performed the conceptual design of the study, supervision of the whole study, revised the manuscript and provide intellectual content, also participated in the acquisition of funding; GR conceived and supervised the whole study, contributed in the interpretation of the data, gave intellectual support, contributed in the manuscript writing and helped in the acquisition of funding. All authors have given final approval of the version to be published.

## Pre-publication history

The pre-publication history for this paper can be accessed here:

http://www.biomedcentral.com/1471-2407/14/529/prepub

## Supplementary Material

Additional file 1 **Haematoxylin and eosin staining images from tissue samples used in the present study.** Sections from FFPE tissue blocks stained with haematoxylin and eosin. **A**, **B**: correspond to samples from normal healthy mammary tissue. **C**, **D**: represent the mammary tumour from young women. **E**, **F**: show breast tumour tissue obtained from older women. Click here for file

Additional file 2 **List and p-values of miRNAs significantly differences in expression between BCVY and BC.** Significantly different expressed miRNAs among two sample groups: young women in breast cancer (BCVY, younger than 35 years old) and older than 65 years women with breast cancer (BC). P-values and FDR p-values (corrected for Benjamini and Hochberg’s False Discovery Rate for multiple comparisons) were obtained performing a *t*-test with 200000 permutations. Nomenclature of miRNAs belong to miRBase v.15. Click here for file

Additional file 3 **List and p-values of miRNAs significantly differences in expression between BCVY and BC.** Significantly different expressed miRNAs among two sample groups: young women in breast cancer (BCVY, younger than 35 years old) and older than 65 years women with breast cancer (BC), headed by Age FDR (from Additional file 1: Table S1), the association of miRNA was further analyzed correcting for tumour characteristics (grade, tumour size, Ki67 and nodal status) to assess whether there are any confounding factor. FDR p-values are corrected for Benjamini and Hochberg’s False Discovery Rate for multiple comparisons and were obtained performing a *t*-test with 200000 permutations. Nomenclature of miRNAs belong to miRBase v.15. Click here for file

Additional file 4 **MicroRNA selected for this study from miRNAs significantly associated with young women’s breast cancer in discovery set and its validation.** Clusters were constructed using Average hierarchical clustering method. Statistics of the discovery set were calculated via *t*-test. FDR stands for False Discovery rate multitesting corrected p-value. Only miRNAs remarked in bold were validated on an independent set of patients. Fold change of the validation set were calculated using ΔΔCt method and p-values were estimated via ANOVA test comparing the age groups and adjusted for multiple testing using Tukey’s method. Bold denotes significant associated results in validation set. Click here for file

Additional file 5 **Putative pathways enriched with the miRNAs included in the sub-node 15 and 63.** Code between brackets refers to the pathway code on KEGG, hsa means *homo sapiens*. FDR refers to p-value adjusted by False Discovery Rate. 20 miRNAs were included in a pathway enrichment analysis and obtained the genes union results. Click here for file

Additional file 6 **Information about miRNAs selected for validation.** Information of the miRNAs selected for validation, including 3p or 5p form (3’ or 5’), MIMAT ID, family and sequence of the mature miRNA. “*” refers to the minor form of the miRNA, according to miRBase v.15 nomenclature. Click here for file
