# Supplementary figures and images for: MicroRNA profile in very young women with breast cancer
Source: BMC Cancer. 2014 Jul 21;14:529. doi: 10.1186/1471-2407-14-529 (PMC4223555; doi:10.1186/1471-2407-14-529)

A

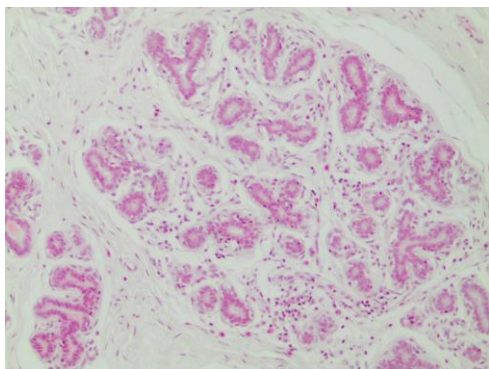

B

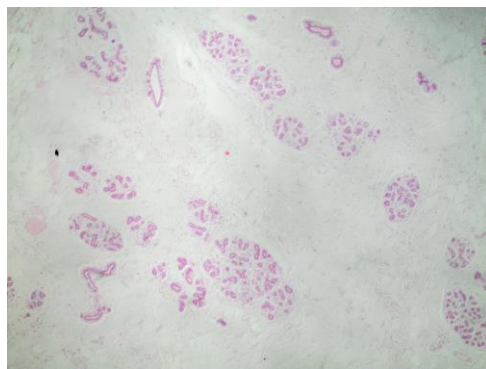

C

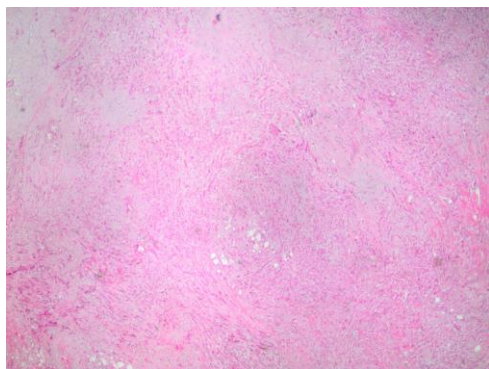

D

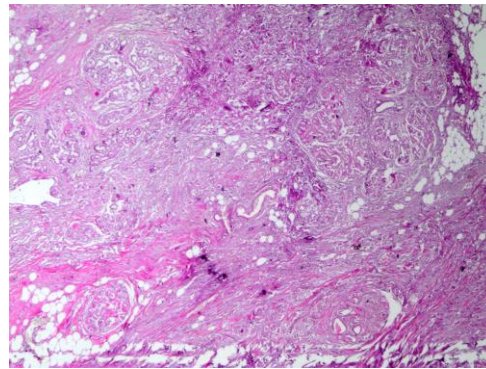

E

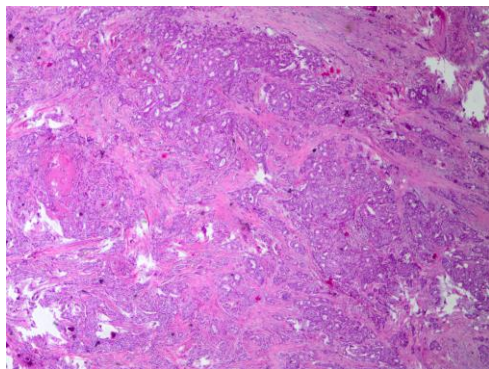

F

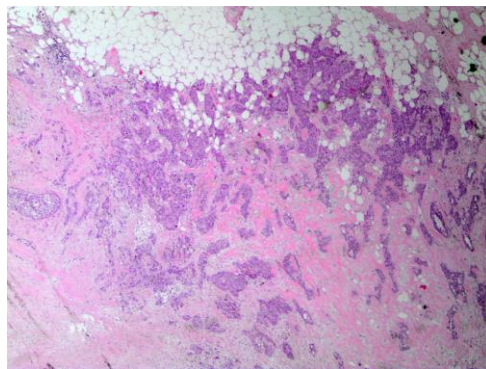

Supplement: Additional file 1 — Haematoxylin and eosin staining images from tissue samples used in the present study. Sections from FFPE tissue blocks stained with haematoxylin and eosin. A, B: correspond to samples from normal healthy mammary tissue. C, D: represent the mammary tumour from young women. E, F: show breast tumour tissue obtained from older women. [file 1471-2407-14-529-S1.pdf]
