# Supplementary material for: MicroRNA profile in very young women with breast cancer
Source: BMC Cancer. 2014 Jul 21;14:529. doi: 10.1186/1471-2407-14-529 (PMC4223555; doi:10.1186/1471-2407-14-529)
Supplement: Additional file 2 — List and p-values of miRNAs significantly differences in expression between BCVY and BC. Significantly different expressed miRNAs among two sample groups: young women in breast cancer (BCVY, younger than 35 years old) and older than 65 years women with breast cancer (BC). P-values and FDR p-values (corrected for Benjamini and Hochberg’s False Discovery Rate for multiple comparisons) were obtained performing a t-test with 200000 permutations. Nomenclature of miRNAs belong to miRBase v.15. [file 1471-2407-14-529-S2.pdf]

Additional File 2.

Title: List and p-values of miRNAs significantly differences in expression between BCVY and BC.

Description: Significantly different expressed miRNAs among two sample groups: young women in breast cancer (BCVY, younger than 35 years old) and older than 65 years women with breast cancer (BC). P-values and FDR p-values (corrected for Benjamini and Hochberg's False Discovery Rate for multiple comparisons) were obtained performing a t-test with 200000 permutations. Nomenclature of miRNAs belong to miRBase v.15.

Additional File 2. List and p-values of miRNAs significantly differences in expression between BCVY and BC.

#	Affymetrix miRNA ID	p-value	FDR	Fold change	Chromosome
1	hsa-miR-3196	5.00x10-6	1.39x10-4	6.745	20
2	hsa-miR-762	5.00x10-6	1.39x10-4	6.646	16
3	hsa-miR-939	5.00x10-6	1.39x10-4	6.586	8
4	hsa-miR-149-star	5.00x10-6	1.39x10-4	6.423	2
5	hsa-miR-1228-star	5.00x10-6	1.39x10-4	6.016	12
6	hsa-miR-1275	5.00x10-6	1.39x10-4	5.887	6
7	hsa-miR-132	5.00x10-6	1.39x10-4	-5.572	17
8	hsa-miR-1908	5.00x10-6	1.39x10-4	5.411	11
9	hsa-miR-28-3p	5.00x10-6	1.39x10-4	-4.425	3
10	hsa-miR-1909	1.00x10-5	1.93x10-4	6.427	19
11	hsa-miR-4299	1.00x10-5	1.93x10-4	5.776	11
12	hsa-miR-3197	1.00x10-5	1.93x10-4	5.768	21
13	hsa-miR-3141	1.00x10-5	1.93x10-4	5.295	5
14	v11_hsa-miR-923	1.50x10-5	2.69x10-4	5.672	17
15	hsa-miR-92b-star	2.00x10-5	3.35x10-4	4.981	1
16	hsa-miR-1202	2.50x10-5	3.69x10-4	5.226	6
17	hsa-miR-125a-5p	2.50x10-5	3.69x10-4	-4.764	19
18	hsa-miR-3175	3.00x10-5	4.18x10-4	4.867	15
19	hsa-miR-23b	5.00x10-5	6.00x10-4	-4.541	9
20	hsa-miR-3162	5.50x10-5	6.00x10-4	5.121	11
21	hsa-miR-1224-5p	5.50x10-5	6.00x10-4	5.000	3
22	hsa-miR-150-star	5.50x10-5	6.00x10-4	4.841	19
23	hsa-miR-638	5.50x10-5	6.00x10-4	4.691	19
24	hsa-miR-1308	6.50x10-5	6.80x10-4	4.592	X
25	hsa-miR-92b	7.00x10-5	7.03x10-4	-4.605	1
26	hsa-miR-1268	7.50x10-5	7.24x10-4	5.013	15
27	hsa-miR-1225-5p	8.00x10-5	7.44x10-4	4.933	16
28	hsa-miR-1207-5p	8.50x10-5	7.62x10-4	4.861	8
29	hsa-miR-27b	1.20x10-4	1.04x10-3	-3.637	9
30	hsa-miR-23a	1.30x10-4	1.09x10-3	-4.478	19
31	hsa-miR-602	1.35x10-4	1.09x10-3	4.084	9
32	hsa-miR-2861	1.50x10-4	1.18x10-3	4.355	9
33	hsa-miR-663	1.70x10-4	1.29x10-3	4.036	20
34	hsa-miR-4281	1.85x10-4	1.37x10-3	4.608	5
35	hsa-miR-665	2.10x10-4	1.51x10-3	4.471	14
36	hsa-miR-30c-1	3.90x10-4	2.72x10-3	-3.424	1
37	hsa-miR-205	4.70x10-4	3.14x10-3	-3.334	1
38	hsa-miR-574-3p	4.75x10-4	3.14x10-3	-4.306	4
39	hsa-miR-1915	5.15x10-4	3.31x10-3	3.836	10
40	hsa-miR-378b	6.40x10-4	4.02x10-3	3.704	3
41	hsa-miR-149	6.70x10-4	4.10x10-3	-4.026	2
42	hsa-miR-26a	6.95x10-4	4.15x10-3	-3.771	10
43	hsa-miR-1973	7.20x10-4	4.20x10-3	3.948	4
44	hsa-miR-148a	8.30x10-4	4.73x10-3	-3.884	7
45	hp_hsa-mir-1224	8.50x10-4	4.74x10-3	3.638	3
46	hsa-miR-183	9.70x10-4	5.29x10-3	-3.907	7
47	hsa-miR-30a-star	1.21x10-3	6.30x10-3	-3.776	6
48	hsa-miR-4270	1.23x10-3	6.30x10-3	3.801	3

49	hsa-miR-28-5p	1.65x10-3	8.31x10-3	-3.137	3
50	hp_hsa-mir-3180-1	1.70x10-3	8.37x10-3	3.426	16
51	hsa-miR-494	1.79x10-3	8.66x10-3	3.459	14
52	hsa-miR-30e-star	1.88x10-3	8.93x10-3	-3.490	1
53	hsa-miR-708	2.02x10-3	9.41x10-3	-3.492	11
54	hsa-miR-22	2.11x10-3	9.65x10-3	-2.446	17
55	hsa-miR-548a-3p	2.25x10-3	1.01x10-2	3.526	22
56	hsa-let-7b	2.37x10-3	1.04x10-2	-3.116	22
57	hsa-miR-4284	2.39x10-3	1.04x10-2	3.143	7
58	hsa-miR-181d	2.73x10-3	1.16x10-2	-3.500	19
59	hsa-miR-3180-3p	2.86x10-3	1.19x10-2	3.326	10
60	hsa-miR-500-star	2.89x10-3	1.19x10-2	-3.287	X
61	hp_hsa-mir-3180-3	3.56x10-3	1.42x10-2	3.281	10
62	hsa-miR-455-3p	3.57x10-3	1.42x10-2	-3.159	9
63	hsa-miR-195	4.52x10-3	1.77x10-2	-2.457	17
64	hsa-miR-1180	5.10x10-3	1.97x10-2	-3.114	17
65	v11_hsa-miR-768-3p	5.52x10-3	2.08x10-2	2.938	16
66	hsa-miR-139-5p	5.55x10-3	2.08x10-2	-3.131	11
67	hsa-miR-152	6.01x10-3	2.22x10-2	-2.949	17
68	hsa-miR-100	6.66x10-3	2.42x10-2	-2.801	11
69	hsa-miR-1972	6.83x10-3	2.45x10-2	2.972	16
70	hsa-miR-29b-2-star	6.96x10-3	2.46x10-2	-2.988	1
71	hsa-miR-1469	7.73x10-3	2.70x10-2	2.771	15
72	hsa-miR-720	8.34x10-3	2.87x10-2	2.897	3
73	hsa-miR-486-5p	8.81x10-3	2.92x10-2	-2.914	8
74	hsa-miR-181a-2-star	8.83x10-3	2.92x10-2	-2.921	9
75	hsa-miR-3178	8.84x10-3	2.92x10-2	2.800	16
76	hsa-miR-361-5p	9.23x10-3	3.01x10-2	-2.862	X
77	hsa-miR-1246	1.01x10-2	3.27x10-2	2.739	2
78	hsa-miR-342-3p	1.13x10-2	3.58x10-2	-2.777	14
79	hsa-miR-339-5p	1.24x10-2	3.89x10-2	-2.729	7
80	hsa-miR-500	1.28x10-2	3.96x10-2	-2.723	X
81	hsa-miR-502-3p	1.31x10-2	4.00x10-2	-2.721	X
82	hsa-miR-29c	1.33x10-2	4.03x10-2	-2.740	1
83	hsa-miR-422a	1.36x10-2	4.07x10-2	-2.686	15
84	hsa-miR-99b-star	1.40x10-2	4.14x10-2	-2.672	19
85	hsa-miR-181a-1	1.46x10-2	4.23x10-2	-2.630	1
86	hsa-miR-26b	1.47x10-2	4.23x10-2	-2.561	2
87	hsa-miR-99b	1.51x10-2	4.31x10-2	-2.594	19
88	hsa-miR-489	1.53x10-2	4.31x10-2	-2.602	7
89	hsa-miR-182	1.60x10-2	4.36x10-2	-2.508	7
90	hsa-miR-409-3p	1.60x10-2	4.36x10-2	-2.624	14
91	hsa-miR-675	1.61x10-2	4.36x10-2	2.604	11
92	hsa-miR-885-3p	1.63x10-2	4.36x10-2	2.641	3
93	hsa-miR-324-3p	1.63x10-2	4.36x10-2	-2.611	17
94	hsa-miR-1274b	1.66x10-2	4.39x10-2	2.567	19
95	hsa-miR-30c-2-star	1.88x10-2	4.91x10-2	-2.564	6
96	hsa-miR-92a	1.93x10-2	5.00x10-2	-2.547	X

Name of the miRNAs corresponds to miRBase v.15 nomenclature, used in Affymetrix array. “v-11” refers to miRBase version 11. P-value was obtained by t-test assessing the differences between BCVY patients (younger than 35 years old) and older than 65 years BC patients. FDR stands for the p-value adjusted by False Discovery Rate. # is the order number according to the signification value.
