# Supplementary material for: MicroRNA profile in very young women with breast cancer
Source: BMC Cancer. 2014 Jul 21;14:529. doi: 10.1186/1471-2407-14-529 (PMC4223555; doi:10.1186/1471-2407-14-529)
Supplement: Additional file 3 — List and p-values of miRNAs significantly differences in expression between BCVY and BC. Significantly different expressed miRNAs among two sample groups: young women in breast cancer (BCVY, younger than 35 years old) and older than 65 years women with breast cancer (BC), headed by Age FDR (from Additional file 1: Table S1), the association of miRNA was further analyzed correcting for tumour characteristics (grade, tumour size, Ki67 and nodal status) to assess whether there are any confounding factor. FDR p-values are corrected for Benjamini and Hochberg’s False Discovery Rate for multiple comparisons and were obtained performing a t-test with 200000 permutations. Nomenclature of miRNAs belong to miRBase v.15. [file 1471-2407-14-529-S3.pdf]

**Additional File 3.**

**Title:** List and p-values of miRNAs significantly differences in expression between BCVY and BC.

**Description:** Significantly different expressed miRNAs among two sample groups: young women in breast cancer (BCVY, younger than 35 years old) and older than 65 years women with breast cancer (BC), headed by Age FDR (from Supplementary Table 1), the association of miRNA was further analyzed correcting for tumour characteristics (grade, tumour size, Ki67 and nodal status) to assess whether there are any confounding factor. FDR p-values are corrected for Benjamini and Hochberg's False Discovery Rate for multiple comparisons and were obtained performing a t-test with 200000 permutations. Nomenclature of miRNAs belong to miRBase v.15.

Additional File 3. P-values of miRNAs corrected by tumour characteristics showing differences in miRNA expression between BCVY and BC.

| # | miRNA ID | Age FDR | FDR corrected by | | | |
|---|---|---|---|---|---|---|
| | | | Grade | Tumour size | Ki67 | Nodal status |
| 1 | hsa-miR-3196 | 1.39x10-4 | 1.79x10-4 | 1.39x10-4 | 1.39x10-4 | 1.14x10-4 |
| 2 | hsa-miR-762 | 1.39x10-4 | 1.79x10-4 | 1.39x10-4 | 1.39x10-4 | 1.14x10-4 |
| 3 | hsa-miR-939 | 1.39x10-4 | 1.79x10-4 | 1.39x10-4 | 1.39x10-4 | 1.14x10-4 |
| 4 | hsa-miR-149-star | 1.39x10-4 | 1.79x10-4 | 1.39x10-4 | 1.39x10-4 | 1.14x10-4 |
| 5 | hsa-miR-1228-star | 1.39x10-4 | 1.79x10-4 | 1.39x10-4 | 1.39x10-4 | 1.14x10-4 |
| 6 | hsa-miR-1275 | 1.39x10-4 | 1.79x10-4 | 1.39x10-4 | 1.79x10-4 | 1.14x10-4 |
| 7 | hsa-miR-132 | 1.39x10-4 | 2.51x10-4 | 1.39x10-4 | 1.79x10-4 | 1.14x10-4 |
| 8 | hsa-miR-1908 | 1.39x10-4 | 1.79x10-4 | 1.39x10-4 | 1.39x10-4 | 1.14x10-4 |
| 9 | hsa-miR-28-3p | 1.39x10-4 | 3.59x10-4 | 1.39x10-4 | 1.39x10-4 | 2.51x10-4 |
| 10 | hsa-miR-1909 | 1.93x10-4 | 3.14x10-4 | 2.09x10-4 | 1.39x10-4 | 1.14x10-4 |
| 11 | hsa-miR-4299 | 1.93x10-4 | 4.18x10-4 | 2.69x10-4 | 1.79x10-4 | 1.14x10-4 |
| 12 | hsa-miR-3197 | 1.93x10-4 | 3.59x10-4 | 2.69x10-4 | 1.79x10-4 | 1.93x10-4 |
| 13 | hsa-miR-3141 | 1.93x10-4 | 2.51x10-4 | 2.09x10-4 | 1.79x10-4 | 1.93x10-4 |
| 14 | v11_hsa-miR-923 | 2.69x10-4 | 4.71x10-4 | 4.18x10-4 | 4.62x10-4 | 1.14x10-4 |
| 15 | hsa-miR-92b-star | 3.35x10-4 | 3.14x10-4 | 2.09x10-4 | 1.39x10-4 | 2.51x10-4 |
| 16 | hsa-miR-1202 | 3.69x10-4 | 7.26x10-4 | 7.09x10-4 | 5.97x10-4 | 3.49x10-4 |
| 17 | hsa-miR-125a-5p | 3.69x10-4 | 5.90x10-4 | 8.51x10-4 | 6.92x10-4 | 4.39x10-4 |
| 18 | hsa-miR-3175 | 4.18x10-4 | 2.51x10-4 | 5.65x10-4 | 6.27x10-4 | 3.49x10-4 |
| 19 | hsa-miR-23b | 6.00x10-4 | 1.21x10-3 | 5.58x10-4 | 6.92x10-4 | 5.52x10-4 |
| 20 | hsa-miR-3162 | 6.00x10-4 | 8.53x10-4 | 4.71x10-4 | 6.92x10-4 | 5.13x10-4 |
| 21 | hsa-miR-1224-5p | 6.00x10-4 | 6.97x10-4 | 7.84x10-4 | 6.92x10-4 | 3.49x10-4 |
| 22 | hsa-miR-150-star | 6.00x10-4 | 8.53x10-4 | 5.97x10-4 | 5.97x10-4 | 4.39x10-4 |
| 23 | hsa-miR-638 | 6.00x10-4 | 8.53x10-4 | 8.03x10-4 | 4.43x10-4 | 5.52x10-4 |
| 24 | hsa-miR-1308 | 6.80x10-4 | 1.43x10-3 | 8.36x10-4 | 4.43x10-4 | 5.79x10-4 |
| 25 | hsa-miR-92b | 7.03x10-4 | 1.21x10-3 | 8.20x10-4 | 6.27x10-4 | 1.00x10-3 |
| 26 | hsa-miR-1268 | 7.24x10-4 | 8.53x10-4 | 6.27x10-4 | 6.27x10-4 | 5.13x10-4 |
| 27 | hsa-miR-1225-5p | 7.44x10-4 | 8.16x10-4 | 5.58x10-4 | 3.35x10-4 | 5.52x10-4 |
| 28 | hsa-miR-1207-5p | 7.62x10-4 | 8.53x10-4 | 5.65x10-4 | 9.89x10-4 | 7.90x10-4 |
| 29 | hsa-miR-27b | 1.04x10-3 | 2.93x10-3 | 1.12x10-3 | 7.29x10-4 | 1.78x10-3 |
| 30 | hsa-miR-23a | 1.09x10-3 | 1.51x10-3 | 1.42x10-3 | 9.96x10-4 | 1.00x10-3 |
| 31 | hsa-miR-602 | 1.09x10-3 | 2.93x10-3 | 1.42x10-3 | 1.32x10-3 | 1.66x10-3 |
| 32 | hsa-miR-2861 | 1.18x10-3 | 2.09x10-3 | 1.75x10-3 | 1.49x10-3 | 1.09x10-3 |
| 33 | hsa-miR-663 | 1.29x10-3 | 2.47x10-3 | 1.83x10-3 | 9.89x10-4 | 1.78x10-3 |
| 34 | hsa-miR-4281 | 1.37x10-3 | 2.10x10-3 | 1.69x10-3 | 1.49x10-3 | 1.25x10-3 |
| 35 | hsa-miR-665 | 1.51x10-3 | 2.16x10-3 | 1.77x10-3 | 1.64x10-3 | 1.29x10-3 |
| 36 | hsa-miR-30c-1 | 2.72x10-3 | 7.53x10-3 | 4.16x10-3 | 7.29x10-4 | 4.28x10-3 |
| 37 | hsa-miR-205 | 3.14x10-3 | 8.68x10-3 | 5.47x10-3 | 1.32x10-3 | 2.61x10-3 |
| 38 | hsa-miR-574-3p | 3.14x10-3 | 5.63x10-3 | 5.31x10-3 | 3.12x10-3 | 1.00x10-3 |
| 39 | hsa-miR-1915 | 3.31x10-3 | 6.73x10-3 | 4.90x10-3 | 3.44x10-3 | 3.92x10-3 |
| 40 | hsa-miR-378b | 4.02x10-3 | 3.60x10-3 | 2.65x10-3 | 8.37x10-3 | 4.28x10-3 |
| 41 | hsa-miR-149 | 4.10x10-3 | 2.93x10-3 | 6.69x10-3 | 3.38x10-3 | 2.99x10-3 |

| | | | | | | |
|---|---|---|---|---|---|---|
| 42 | hsa-miR-26a | 4.15x10-3 | 6.18x10-3 | 4.90x10-3 | 1.82x10-3 | 4.01x10-3 |
| 43 | hsa-miR-1973 | 4.20x10-3 | 7.53x10-3 | 6.75x10-3 | 5.87x10-3 | 4.88x10-3 |
| 44 | hsa-miR-148a | 4.73x10-3 | 6.51x10-3 | 4.50x10-3 | 4.78x10-3 | 6.43x10-3 |
| 45 | hp_hsa-mir-1224 | 4.74x10-3 | 5.02x10-3 | 6.75x10-3 | 4.77x10-3 | 4.21x10-3 |
| 46 | hsa-miR-183 | 5.29x10-3 | 4.89x10-3 | 8.61x10-3 | 5.22x10-3 | 5.58x10-3 |
| 47 | hsa-miR-30a-star | 6.30x10-3 | 7.47x10-3 | 6.68x10-3 | 4.62x10-4 | 4.32x10-3 |
| 48 | hsa-miR-4270 | 6.30x10-3 | 8.26x10-3 | 7.43x10-3 | 1.03x10-2 | 5.88x10-3 |
| 49 | hsa-miR-28-5p | 8.31x10-3 | 1.67x10-2 | 3.90x10-3 | 7.20x10-3 | 8.49x10-3 |
| 50 | hp_hsa-mir-3180-1 | 8.37x10-3 | 1.07x10-2 | 1.04x10-2 | 5.18x10-3 | 8.23x10-3 |
| 51 | hsa-miR-494 | 8.66x10-3 | 7.53x10-3 | 9.78x10-3 | 1.22x10-2 | 8.64x10-3 |
| 52 | hsa-miR-30e-star | 8.93x10-3 | 1.65x10-2 | 1.10x10-2 | 8.37x10-3 | 8.71x10-3 |
| 53 | hsa-miR-708 | 9.41x10-3 | 1.65x10-2 | 6.69x10-3 | 7.86x10-3 | 8.49x10-3 |
| 54 | hsa-miR-22 | 9.65x10-3 | 3.56x10-2 | 1.43x10-2 | 6.56x10-3 | 2.07x10-2 |
| 55 | hsa-miR-548a-3p | 1.01x10-2 | 1.80x10-2 | 1.04x10-2 | 6.63x10-3 | 1.11x10-2 |
| 56 | hsa-let-7b | 1.04x10-2 | 2.12x10-2 | 1.96x10-2 | 6.92x10-4 | 1.10x10-2 |
| 57 | hsa-miR-4284 | 1.04x10-2 | 1.13x10-2 | 1.49x10-2 | 2.09x10-2 | 1.11x10-2 |
| 58 | hsa-miR-181d | 1.16x10-2 | 1.65x10-2 | 1.30x10-2 | 8.37x10-3 | 1.23x10-2 |
| 59 | hsa-miR-3180-3p | 1.19x10-2 | 1.29x10-2 | 1.42x10-2 | 8.18x10-3 | 1.25x10-2 |
| 60 | hsa-miR-500-star | 1.19x10-2 | 1.90x10-2 | 1.08x10-2 | 1.35x10-2 | 1.20x10-2 |
| 61 | hp_hsa-mir-3180-3 | 1.42x10-2 | 1.90x10-2 | 1.70x10-2 | 1.48x10-2 | 1.47x10-2 |
| 62 | hsa-miR-455-3p | 1.42x10-2 | 1.33x10-2 | 4.67x10-3 | 1.45x10-2 | 1.30x10-2 |
| 63 | hsa-miR-195 | 1.77x10-2 | 4.89x10-2 | 2.68x10-2 | 4.35x10-3 | 2.74x10-2 |
| 64 | hsa-miR-1180 | 1.97x10-2 | 2.01x10-2 | 1.98x10-2 | 1.26x10-2 | 1.47x10-2 |
| 65 | v11_hsa-miR-768-3p | 2.08x10-2 | 3.26x10-3 | 1.22x10-2 | 2.77x10-2 | 1.47x10-2 |
| 66 | hsa-miR-139-5p | 2.08x10-2 | 2.28x10-2 | 3.06x10-2 | 1.72x10-2 | 2.03x10-2 |
| 67 | hsa-miR-152 | 2.22x10-2 | 4.75x10-2 | 1.27x10-2 | 2.02x10-2 | 2.33x10-2 |
| 68 | hsa-miR-100 | 2.42x10-2 | 5.61x10-2 | 2.92x10-2 | 2.57x10-2 | 2.84x10-2 |
| 69 | hsa-miR-1972 | 2.45x10-2 | 2.93x10-2 | 2.83x10-2 | 1.32x10-2 | 2.84x10-2 |
| 70 | hsa-miR-29b-2-star | 2.46x10-2 | 4.06x10-2 | 3.69x10-2 | 4.28x10-3 | 2.54x10-2 |
| 71 | hsa-miR-1469 | 2.70x10-2 | 4.40x10-2 | 3.31x10-2 | 2.09x10-2 | 2.84x10-2 |
| 72 | hsa-miR-720 | 2.87x10-2 | 1.05x10-2 | 4.92x10-2 | 3.41x10-2 | 2.84x10-2 |
| 73 | hsa-miR-486-5p | 2.92x10-2 | 4.40x10-2 | 5.12x10-2 | 4.11x10-2 | 2.89x10-2 |
| 74 | hsa-miR-181a-2-star | 2.92x10-2 | 4.25x10-2 | 1.95x10-2 | 2.88x10-2 | 2.84x10-2 |
| 75 | hsa-miR-3178 | 2.92x10-2 | 4.58x10-2 | 3.58x10-2 | 1.12x10-2 | 2.84x10-2 |
| 76 | hsa-miR-361-5p | 3.01x10-2 | 5.06x10-2 | 5.27x10-2 | 7.32x10-3 | 2.86x10-2 |
| 77 | hsa-miR-1246 | 3.27x10-2 | 4.90x10-2 | 4.37x10-2 | 2.14x10-2 | 1.97x10-2 |
| 78 | hsa-miR-342-3p | 3.58x10-2 | 4.75x10-2 | 4.67x10-2 | 7.86x10-3 | 3.39x10-2 |
| 79 | hsa-miR-339-5p | 3.89x10-2 | 4.90x10-2 | 4.68x10-2 | 2.41x10-2 | 3.15x10-2 |
| 80 | hsa-miR-500 | 3.96x10-2 | 4.58x10-2 | 3.01x10-2 | 4.57x10-2 | 3.39x10-2 |
| 81 | hsa-miR-502-3p | 4.00x10-2 | 4.75x10-2 | 3.90x10-2 | 4.11x10-2 | 3.39x10-2 |
| 82 | hsa-miR-29c | 4.03x10-2 | 4.75x10-2 | 4.70x10-2 | 1.26x10-2 | 3.63x10-2 |
| 83 | hsa-miR-422a | 4.07x10-2 | 4.75x10-2 | 4.70x10-2 | 3.76x10-2 | 4.41x10-2 |
| 84 | hsa-miR-99b-star | 4.14x10-2 | 3.03x10-2 | 4.07x10-2 | 4.23x10-2 | 3.39x10-2 |
| 85 | hsa-miR-181a-1 | 4.23x10-2 | 5.56x10-2 | 4.92x10-2 | 4.43x10-2 | 3.27x10-2 |
| 86 | hsa-miR-26b | 4.23x10-2 | 4.42x10-2 | 4.70x10-2 | 4.04x10-2 | 4.65x10-2 |
| 87 | hsa-miR-99b | 4.31x10-2 | 4.42x10-2 | 7.69x10-2 | 3.60x10-2 | 3.63x10-2 |
| 88 | hsa-miR-489 | 4.31x10-2 | 4.75x10-2 | 5.85x10-2 | 2.77x10-2 | 4.41x10-2 |
| 89 | hsa-miR-182 | 4.36x10-2 | 4.75x10-2 | 4.92x10-2 | 2.73x10-2 | 4.78x10-2 |
| 90 | hsa-miR-409-3p | 4.36x10-2 | 6.52x10-2 | 6.12x10-2 | 4.77x10-2 | 4.38x10-2 |
| 91 | hsa-miR-675 | 4.36x10-2 | 5.53x10-2 | 5.03x10-2 | 4.11x10-2 | 4.38x10-2 |
| 92 | hsa-miR-885-3p | 4.36x10-2 | 4.75x10-2 | 2.90x10-2 | 3.27x10-2 | 2.93x10-2 |
| 93 | hsa-miR-324-3p | 4.36x10-2 | 6.71x10-2 | 5.23x10-2 | 2.86x10-2 | 4.51x10-2 |
| 94 | hsa-miR-1274b | 4.39x10-2 | 2.28x10-2 | 7.24x10-2 | 5.47x10-2 | 4.41x10-2 |
| 95 | hsa-miR-30c-2-star | 4.91x10-2 | 6.62x10-2 | 7.22x10-2 | 2.41x10-2 | 5.21x10-2 |
| 96 | hsa-miR-92a | 5.00x10-2 | 3.00x10-2 | 4.92x10-2 | 6.96x10-2 | 4.80x10-2 |

Name of the miRNAs corresponds to miRBase v.15 nomenclature, used in Affymetrix array. “v-11” refers to miRBase version 11. P-value was obtained by t-test assessing the differences between groups of samples. FDR stands for the p-value adjusted by False Discovery Rate. # is the order number according to the signification value. Age FDR shows the original p-values considering age as the only factor. Grade FDR, Tumour size FDR, Ki67 FDR and Nodal status FDR refer to the p-values corrected by each tumour characteristic respectively.
