# Supplementary material for: MicroRNA profile in very young women with breast cancer
Source: BMC Cancer. 2014 Jul 21;14:529. doi: 10.1186/1471-2407-14-529 (PMC4223555; doi:10.1186/1471-2407-14-529)
Supplement: Additional file 4 — MicroRNA selected for this study from miRNAs significantly associated with young women’s breast cancer in discovery set and its validation. Clusters were constructed using Average hierarchical clustering method. Statistics of the discovery set were calculated via t-test. FDR stands for False Discovery rate multitesting corrected p-value. Only miRNAs remarked in bold were validated on an independent set of patients. Fold change of the validation set were calculated using ΔΔCt method and p-values were estimated via ANOVA test comparing the age groups and adjusted for multiple testing using Tukey’s method. Bold denotes significant associated results in validation set. [file 1471-2407-14-529-S4.pdf]

Additional File 4.

Title: MicroRNA selected for this study from miRNAs significantly associated with young women's breast cancer in discovery set and its validation.

Description: Clusters were constructed using Average hierarchical clustering method. Statistics of the discovery set were calculated via t-test. FDR stands for False Discovery rate multitesting corrected p-value. Only miRNAs remarked in bold were validated on an independent set of patients. Fold change of the validation set were calculated using ΔΔCt method and p-values were estimated via ANOVA test comparing the age groups and adjusted for multiple testing using Tukey's method. Bold denotes significant associated results in validation set.

Additional File 4. MicroRNA selected for this study from miRNAs significantly associated with young women's breast cancer in discovery set and its validation.

		Discovery set (n=37)		Validation set (n=56)
cluster	miR	FDR	Fold change	Adjusted p-value
Node 15	hsa-miR-1275	$1.40x10^{-4}$	5.89	**$2.86x10^{-3}$**
	hsa-miR-1207-5p	$7.60x10^{-4}$	4.86	0.047
	hsa-miR-4281	$1.37x10^{-3}$	4.61	
	hsa-miR-1268	$7.2x10^{-4}$	5.01	
	hsa-miR-663	$1.29x10^{-3}$	4.04	
	hsa-miR-149	$4.10x10^{-3}$	-4.03	0.712
	hsa-miR-762	$1.40x10^{-4}$	6.65	
	hsa-miR-3196	$1.40x10^{-4}$	6.74	**$1.98x10^{-4}$**
	hsa-miR-1908	$1.40x10^{-4}$	5.41	
	hsa-miR-1228*	$1.40x10^{-4}$	6.02	**$4.73 x10^{-5}$**
	hsa-miR-3141	$1.90x10^{-4}$	5.30	
	hsa-miR-92b	$7.00x10^{-4}$	-4.60	**0.012**
Node 63	hsa-miR-139-5p	0.020	-3.13	**0.018**
	hsa-miR-132	$1.40x10^{-4}$	-5.57	0.908
	hsa-miR-379	0.092	-2.14	0.052
	hsa-miR-409-3p	0.043	-2.62	0.107
	hsa-miR-134	0.086	-2.20	
	hsa-miR-127-3p	0.050	-2.53	
	hsa-miR-433	0.093	-2.15	0.132
	hsa-miR-181a-2*	0.029	-2.92	

Clusters were constructed using Average hierarchical clustering method. Statistics of the discovery set were calculated via t-test. FDR stands for False Discovery rate multitesting corrected p-value. Only miRNAs remarked in bold were validated on an independent set of patients. Expression values of the validation set were calculated using ΔΔCt method and p-values were estimated via ANOVA test comparing the age groups and adjusted for multiple testing using Tukey's method. Bold denotes significant associated results in validation set.
