# Supplementary material for: MicroRNA profile in very young women with breast cancer
Source: BMC Cancer. 2014 Jul 21;14:529. doi: 10.1186/1471-2407-14-529 (PMC4223555; doi:10.1186/1471-2407-14-529)
Supplement: Additional file 5 — Putative pathways enriched with the miRNAs included in the sub-node 15 and 63. Code between brackets refers to the pathway code on KEGG, hsa means homo sapiens. FDR refers to p-value adjusted by False Discovery Rate. 20 miRNAs were included in a pathway enrichment analysis and obtained the genes union results. [file 1471-2407-14-529-S5.pdf]

Additional File 5.

Title: Putative pathways enriched with the miRNAs included in the sub-node 15 and 63.

Description: Code between brackets refers to the pathway code on KEGG, hsa means *homo sapiens*. FDR refers to p-value adjusted by False Discovery Rate. 20 miRNAs were included in a pathway enrichment analysis and obtained the genes union results.

Additional File 5. Putative pathways enriched with the miRNAs included in the sub-node 15 and 63

KEGG pathway (hsa)	Number of miR	FDR	Number of genes
ECM-receptor interaction (hsa04512)	14	$1.60x10^{-13}$	33
Endocytosis (hsa04144)	15	$3.17x10^{-7}$	67
Glycosaminoglycan biosynthesis – heparan sulfate (hsa00534)	6	$1.62x10^{-6}$	8
Adherens junction (hsa04520)	13	$3.76x10^{-5}$	26
Circadian rhythm - mammal (hsa04710)	9	$5.88x10^{-5}$	11
Focal adhesion (hsa04510)	16	$5.88x10^{-5}$	67
Axon guidance (hsa04360)	13	$3.92x10^{-4}$	41
Biotin metabolism (hsa00780)	1	$3.97x10^{-4}$	1
Arrhythmogenic right ventricular cardiomyopathy (ARVC) (hsa05412)	13	$1.92x10^{-3}$	26
Long-term depression (hsa04730)	12	$2.19x10^{-2}$	22
Cell adhesion molecules (CAMs) (hsa04514)	13	$2.19x10^{-2}$	34
Dilated cardiomyopathy (hsa05414)	16	$2.19x10^{-2}$	30
Fc gamma R-mediated phagocytosis (hsa04666)	9	$2.96x10^{-2}$	30
Hypertrophic cardiomyopathy (HCM) (hsa05410)	15	$4.54x10^{-2}$	27
MAPK signaling pathway (hsa04010)	16	$4.54x10^{-2}$	7267
Aldosterone-regulated sodium reabsorption (hsa04960)	11	$4.69x10^{-2}$	16
Regulation of actin cytoskeleton (hsa04810)	15	$4.69x10^{-2}$	56

Code between brackets refers to the pathway code on KEGG, hsa means *homo sapiens*. FDR refers to p-value adjusted by False Discovery Rate. 20 miRNAs were included in a pathway enrichment analysis and obtained the genes union results.
