# Supplementary material for: MicroRNA profile in very young women with breast cancer
Source: BMC Cancer. 2014 Jul 21;14:529. doi: 10.1186/1471-2407-14-529 (PMC4223555; doi:10.1186/1471-2407-14-529)
Supplement: Additional file 6 — Information about miRNAs selected for validation. Information of the miRNAs selected for validation, including 3p or 5p form (3’ or 5’), MIMAT ID, family and sequence of the mature miRNA. “*” refers to the minor form of the miRNA, according to miRBase v.15 nomenclature. [file 1471-2407-14-529-S6.pdf]

Additional File 6.

Title: Information about miRNAs selected for validation.

Description: Information of the miRNAs selected for validation, including 3p or 5p form (3' or 5'), MIMAT ID, family and sequence of the mature miRNA. "*" refers to the minor form of the miRNA, according to miRBase v.15 nomenclature.

Additional File 6. Information about miRNAs selected for validation.

miRBase v.18 name	miRBase ID	family	sequence
hsa-miR-92b-3p	MIMAT0003658	mir-25	UAUUGCACUCGUCCCGGCCUC
hsa-miR-92b-5p(*)	MIMAT0004792	mir-25	AGGGACGGGACGCGGUGCAGUG
hsa-miR-132-3p	MIMAT0000426	mir-132	UAACAGUCUACAGCCAUGGUCG
hsa-miR-139-5p	MIMAT0000250	mir-139	UCUACAGUGCACGUGUCUCCAGU
hsa-miR-149-5p	MIMAT0000450	mir-149	UCUGGCUCCGUGUCUUCACUCCC
hsa-miR-149-3p(*)	MIMAT0004609	mir-149	AGGGAGGGACGGGGGCUGUGC
hsa-miR-433	MIMAT0001627	mir-433	AUCAUGAUGGGCUCCUCGGUGU
hsa-miR-409-3p	MIMAT0001639	mir-154	GAAUGUUGCUCGGUGAACCCCU
hsa-miR-379-5p	MIMAT0000733	mir-379	UGGUAGACUAUGGAACGUAGG
hsa-miR-1207-5p	MIMAT0005871	mir-1207	UGGCAGGGAGGCUGGGAGGGG
hsa-miR-1275	MIMAT0005929	mir-1275	GUGGGGGAGAGGCUGUC
hsa-miR-1228-5p(*)	MIMAT0005582	mir-1228	GUGGGCGGGGGCAGGUGUGUG
hsa-miR-3196	MIMAT0015080		CGGGGCGGCAGGGGCCUC
